# Intuitive eating in Greek-Cypriot adults: Influence of gender and body mass

**DOI:** 10.3389/fpsyg.2022.1033720

**Published:** 2022-11-24

**Authors:** Marios Argyrides, Elly Anastasiades

**Affiliations:** Department of Psychology, Neapolis University Pafos, Pafos, Cyprus

**Keywords:** intuitive eating, wellbeing, eating behavior, adaptive eating, body image

## Abstract

**Introduction:**

To date, research on eating behaviors has largely taken a pathological approach. Researchers are increasingly taking a positive approach to explore adaptive eating styles. One such style that has recently received much research attention is intuitive eating. Recent work examining intuitive eating and its relationships with body mass and gender has yielded mixed findings. The current study explored the differential effects of gender and body mass on intuitive eating scores in a sample of Greek-Cypriot adults.

**Method:**

A total of 1,312 adult participants (women *n* = 655; *M*_age_ = 34.49) completed the Intuitive Eating Scale-2 and provided demographic information.

**Results:**

Our analyses revealed that men reported significantly higher intuitive eating scores than women. Body mass was significantly inversely associated with intuitive eating in both men and women. Additionally, a multivariate analysis of variance (MANOVA) indicated significant interaction effects between gender and body mass on intuitive eating. These interaction effects were explored for each of the four subscales of intuitive eating, identifying differential associations for intuitive eating and BMI between men and women.

**Discussion:**

Both gender and body mass are important factors which influence intuitive eating levels in Greek-Cypriot adults. Discussions of how these findings can inform future research, theory and practice are presented.

## Introduction

Until recently, eating behavior research has largely taken a pathology-driven approach, focusing on the description, explanation and prediction of restrictive and disordered eating behavior. While such research has proven invaluable in guiding the diagnosis, prevention and treatment of disordered eating, this approach is limited as it focuses solely on the reduction of eating-related distress and does not take into consideration health-enhancing eating behaviors. More recently, scholars are increasingly taking a positive psychological approach, focusing on adaptive eating behaviors (i.e., those that are guided by the body’s internal physiological cues rather than external situational and socioemotional cues; Resch and Tylka, 2019). This work has elucidated the importance of adaptive eating behaviors not only in protecting against the development of disordered eating, but also in enhancing health and wellbeing (Tylka and Wilcox, 2006; Van Dyke and Drinkwater, 2014; Tylka et al., 2015). An adaptive eating style that has recently gained much research attention has been operationalized as *intuitive eating* (Tribole and Resch, 1995, 2012). Intuitive eating reflects a set of flexible eating behaviors that are guided by internal physiological hunger and satiety signals. Those who eat intuitively trust and depend on their internal cues to drive their eating behavior, avoid labeling foods as prohibited, eat for physical reasons rather than to cope with their emotions, and make food choices that support or enhance their body’s functioning and wellbeing (Tribole and Resch, 2012; Tylka and Kroon Van Diest, 2013; Resch and Tylka, 2019).

Intuitive eating has consistently been found to be closely related to psychosocial factors including facets of negative and positive body image. More specifically, researchers have reported associations for intuitive eating with lower levels of psychological distress, body image dissatisfaction, restrictive/disordered eating, and higher levels of quality of life, body appreciation, self-esteem, self-compassion and wellbeing (e.g., Augustus-Horvath and Tylka, 2011; Tylka et al., 2015; Webb and Hardin, 2016; Homan and Tylka, 2018; Linardon et al., 2020; Anastasiades and Argyrides, 2022; Gödde et al., 2022; Jackson et al., 2022; Teas et al., 2022). These findings have been replicated across a variety of ages, body mass categories, genders and cultural and ethnic groups (for a review, see Bruce and Ricciardelli, 2016; Linardon et al., 2021). Crucially, recent studies using experimental and prospective designs are yielding promising results with intuitive eating being found to lower symptoms of disordered eating and enhance body image, self-esteem, self-compassion, quality of life and psychological wellbeing (Bush et al., 2014; Humphrey et al., 2015; Carbonneau et al., 2017; Wilson et al., 2020; Babbott et al., 2022).

Accumulating empirical work provides strong support for intuitive eating being closely linked to body image. This highlights the importance of exploring the links between intuitive eating and other factors that have been found to play an important role in body image. Two such factors are gender and body mass. A higher body mass is often perceived as unhealthy and undesirable (Pearl et al., 2020). As such, individuals with a higher body mass have reported increased levels of body dissatisfaction and disordered eating (e.g., binge eating and emotional eating), and lower levels of body appreciation (Vartanian and Porter, 2016; Pearl et al., 2020). With regard to gender, there is much evidence suggesting that women face increased societal pressures to conform to unrealistic beauty ideals, with women generally reporting higher levels of body dissatisfaction and lower levels of body appreciation. Even though men also face increasing pressure to conform to ideals (i.e., an athletic, muscular physique; Frederick et al., 2022c), evidence suggests that the pressure and scrutiny women face is both more frequent and more severe (Grogan, 2016; Frederick et al., 2022a). Research exploring the links between intuitive eating and gender and body mass has yielded mixed findings (Linardon et al., 2021). However, a recent meta-analysis found intuitive eating to be (a) consistently negatively related to body mass, and (b) significantly higher in men than in women across a variety of age groups and cultures (Linardon et al., 2021). Despite this, the authors noted limited variability with respect to demographic characteristics (including gender, ethnicity, and culture) across studies examining intuitive eating.

One context in which intuitive eating has rarely been examined is in the Greek-Cypriot population. There are several reasons why intuitive eating is worth examining in this population. For instance, traditionally, the Cypriot diet is a Mediterranean diet, characterized by moderate consumption of seafood, meat and dairy, and high consumption of extra-virgin olive oil, whole grains, olives, legumes, nuts, seeds, and fruits and vegetables (Hidalgo-Mora et al., 2020). There is much research to support the health benefits of consuming a Mediterranean diet including improved life expectancy, quality of life, and lower incidence of chronic disease (Sánchez-Sánchez et al., 2020; Sezaki et al., 2022). In addition, some of the traditional food preparation and consumption practices in the Mediterranean region promote eating as an “embodied” process and may therefore affect intuitive eating behaviors. For instance, eating is largely a socially valued event where food is prepared socially, with care, and the sensory properties of food are acknowledged, discussed and savored (Sutton, 2009).

Surprisingly, however, obesity and overweight rates in Cyprus are among the highest in Europe, and continue to rise, with 33.8% of adults having overweight body mass, and 16.6% of adults having obesity (Eurostat, 2022). In addition, Cyprus has been found to have high levels of weight-related anxiety in relation to other European countries (Argyrides et al., 2019). This might be explained by the Westernization of eating habits with increased consumption of fast-foods (i.e., foods that are high in saturated fats and refined carbohydrates), facilitated by increased accessibility and ease of availability (e.g., using food delivery applications; Argyrides and Kkeli, 2015). Further, there remains a significant lack of public health initiatives and interventions aimed at the promotion of healthy eating behaviors (see Ministry of Health, 2008). This signifies the importance of examining intuitive eating in this population.

### The current study

There is strong support for the clinical utility of intuitive eating in the realm of intervention efforts aimed at the prevention and treatment of disordered eating and obesity. The development of a detailed understanding of how intuitive eating is related to physical and psychosocial factors is essential for the design, implementation, and efficacy of such efforts. As such, the current study aimed to explore differences in intuitive eating scores based on gender and body mass in the Greek-Cypriot population. Based on previous findings (Linardon et al., 2021), we expected that men would report significantly higher intuitive eating scores than women, and that body mass would be significantly inversely associated with intuitive eating in both men and women. In addition, we explored whether the differences in intuitive eating scores across body mass categories differed for men and women (i.e., if there is an interaction effect between body mass and gender on intuitive eating scores).

## Materials and methods

### Measures

#### Demographics

A demographics questionnaire was completed by all participants in which they were asked to report their age, gender, ethnicity, height and weight. Body Mass Index (BMI) was calculated from height and weight as (kg/m^2^).

#### Intuitive eating

To measure intuitive eating, participants completed Intuitive Eating Scale-2 (IES-2; Tylka and Kroon Van Diest, 2013; Greek translation: Giannakou et al., 2022). The 23-item IES-2 comprises 4 subscales which assess the four facets of intuitive eating; Unconditional Permission to Eat (i.e., an individual’s willingness to eat when hungry and a refusal to label certain foods as forbidden; 6 items), Eating for Physical rather than Emotional Reasons (i.e., eating when one is physically hungry rather than to cope with emotional distress; 8 items), Reliance on Hunger and Satiety Cues (i.e., an individual’s trust in their internal hunger and satiety cues and reliance on these cues to guide eating behaviors; 6 items), and Body-Food Choice Congruence (i.e., a tendency to make food choices that honor one’s health and body functioning; 3 items). All items were rated on a 5-point scale ranging from 1 = *strongly disagree*, to 5 = *strongly agree.* An overall subscale score was computed as the mean of all items, with higher scores reflecting greater intuitive eating. Adequate internal consistency and construct validity have been reported for scores on the Greek translation of the IES-2 (Giannakou et al., 2022). For the current sample, the scale was found to be internally consistent (α = 0.87 for the Total Scale, α = 0.72 for Unconditional Permission to Eat, α = 0.90 for Eating for Physical Rather than Emotional Reasons, α = 0.86 for Reliance on Hunger and Satiety Cues, and α = 0.81 for Body-Food Choice Congruence).

### Participants and procedure

The study was carried out in accordance with the principles of the Declaration of Helsinki, and ethical approval was obtained from the Cyprus Bioethics Committee. Data collection took place between May 2022 and July 2022. Participants were recruited *via* advertisements placed on social media websites supplemented using a snowball sampling method. The study was advertised as a study about “eating styles and behaviors.” Those interested in participating were directed to and online questionnaire, hosted by Qualtrics^1^ and completed a pre-screener to determine eligibility for the study. Participants were eligible if (a) they were over 18 years of age (b) their preferred language was Greek, and (c) they were a citizen of Cyprus. Upon meeting the inclusion criteria, participants were provided with further information regarding the study requirements, including that participation was anonymous, voluntary and without remuneration. Provided digital informed consent before completing the online questionnaire with the measures listed above in a pre-randomized order. Attention checks were placed at two points in the questionnaire.

### Data analysis

All data analyses were conducted using IBM SPSS Statistics Version 28. Missing data was managed using listwise deletion. Data were screened to ensure data quality as recommended when using online samples (Burnette et al., 2022; Moeck et al., 2022). This included checking (a) Internet Protocol addresses to identify whether any participant answered the questionnaire more than once (b) responses for age height and weight to identify nonsensical/improbable values and (c) failed responses to the attention checks. The screening identified six participants who entered nonsensical age/height values, and 10 participants who failed the attention checks, who were subsequently removed from the initial sample of *N* = 1,328, reducing the sample to *N* = 1,312.

Following this, data were assessed for normality, linearity, and homoscedasticity; all assumptions were met. Participant characteristics and intergroup differences based on gender and BMI category were assessed *via* examination of analysis of variance (ANOVA) and Chi-squared testing. The intercorrelations of the study variables were examined using a Pearson’s product moment correlation analysis, with *r* values of ≤0.10 being considered to have a small effect, ~0.30, a moderate effect, and ~0.50, a strong effect (based on Cohen, 1992). To examine subgroup differences in intuitive eating scores, a two-way multivariate analysis of variance (MANOVA; Pillai’s trace correction) was conducted with gender and BMI category entered as independent variables and intuitive eating as the dependent variable. Effect sizes were examined, with partial eta-squared values of ≤0.01 being considered to a small effect, ~0.06 a moderate effect, and ~0.14 a strong effect (based on Cohen, 1988). For all analyses, *p* < 0.05 was considered significant.

## Results

### Participant characteristics

The participants of the study were 1,312 Greek Cypriot citizens and residents (women *n* = 655, men *n* = 657), ranging in age from 18 to 70 years (*M* = 34.49, *SD* = 10.90) and in self-reported body mass index (BMI) from 14.53 to 53.22 kg/m^2^ (*M* = 25.37, *SD* = 5.24). As can be seen in Table 1, on average, the men in this sample had significantly higher self-reported BMI than the women [*F*(1, 1,311) = 120.44, *p* < 0.001], and were significantly older than the women [*F*(1, 1,311) = 21.64, *p* < 0.001]. In addition, the distribution of participants across BMI groups differed significantly between men and women, χ^2^(3, 1,312) = 139.04, *p* < 0.001, with women having more participants in the lower BMI groups (i.e., underweight and normal weight), and men having more participants in the higher BMI groups (i.e., overweight and obese). No significant differences were observed for age differences between each of the BMI groups for men and women.

**Table 1:** Sample characteristics.

	Total sample (*N* = 1,312)	Women (*n* = 657)	Men (*n* = 655)	*p*
BMI (kg/m^2^)	25.37 ± 5.24	23.85 ± 5.09	26.89 ± 4.93	**<0.001**
**BMI groups**				
Underweight	*n* = 61 (4.6%)	*n* = 48 (7.3%)	*n* = 13 (2.0%)	*<0.001
Normal weight	*n* = 660 (50.3%)	*n* = 416 (63.3%)	*n* = 244 (37.3%)	
Overweight	*n* = 389 (29.6%)	*n* = 117 (17.8%)	*n* = 272 (41.5%)	
Obese	*n* = 202 (15.4%)	*n* = 76 (11.6%)	*n* = 126 (19.2%)	
Age (years)	34.5 ± 10.9	33.1 ± 10.6	35.9 ± 11.0	**<0.001**
**Age in BMI groups (years)**				
Underweight	27.5 ± 9.1	26.7 ± 7.5	30.4 ± 13.3	0.187
Normal weight	32.8 ± 10.5	32.2 ± 10.0	33.7 ± 11.3	0.074
Overweight	37.1 ± 10.6	37.1 ± 11.9	37.1 ± 10.1	0.993
Obese	37.3 ± 11.2	36.0 ± 11.2	38.1 ± 11.3	0.193

Data are M ± SD or number of participants and percentage. *p*-values are derived from a one-way ANOVA, or *χ^2^-test. *p*-values in bold denote significance (based on alpha = 0.05).

### Inter-correlations

As can be seen in Table 2, for women, the Pearson’s product moment correlation analysis indicated significant moderate inverse associations between BMI and intuitive eating (*r* = −0.35, *p* < 0.001), as well as all four of the subscales of intuitive eating. Similar results were observed for men, with moderate inverse associations for BMI and intuitive eating (*r* = −0.32, *p* < 0.001), as well as all the subscales of intuitive eating besides unconditional permission to eat, which did not reach significance.

**Table 2:** Intercorrelations for study variables.

Variable	1	2	3	4	5	6
Body mass index	—	**−0.35** ^**^	**−0.12** ^**^	**−0.35** ^**^	**−0.26** ^**^	**−0.15** ^**^
Intuitive eating	0.32^**^	—	**0.48** ^**^	**0.88** ^**^	**0.82** ^**^	**0.36** ^**^
UPE	−0.01	0.40^**^	—	**0.20** ^**^	**0.28** ^**^	**−0.26** ^**^
EPRTE	−0.31^**^	0.82^**^	0.03	—	**0.58** ^**^	**0.29** ^**^
ROHSC	−0.21^**^	0.72^**^	0.22^**^	0.33^**^	—	**0.30** ^**^
BFCC	−0.17^**^	0.33^**^	−0.36^**^	0.29^**^	0.19^**^	—

*N* = 1,312. UPE, unconditional permission to eat; EPRTE, eating for physical rather than emotional reasons; ROHSC, reliance on hunger and satiety cues; BFCC, body-food choice congruence. Values in bold represent women.

* *p* < 0.05.

** *p* < 0.001.

### Comparison by gender and BMI category

**Figure 1:**
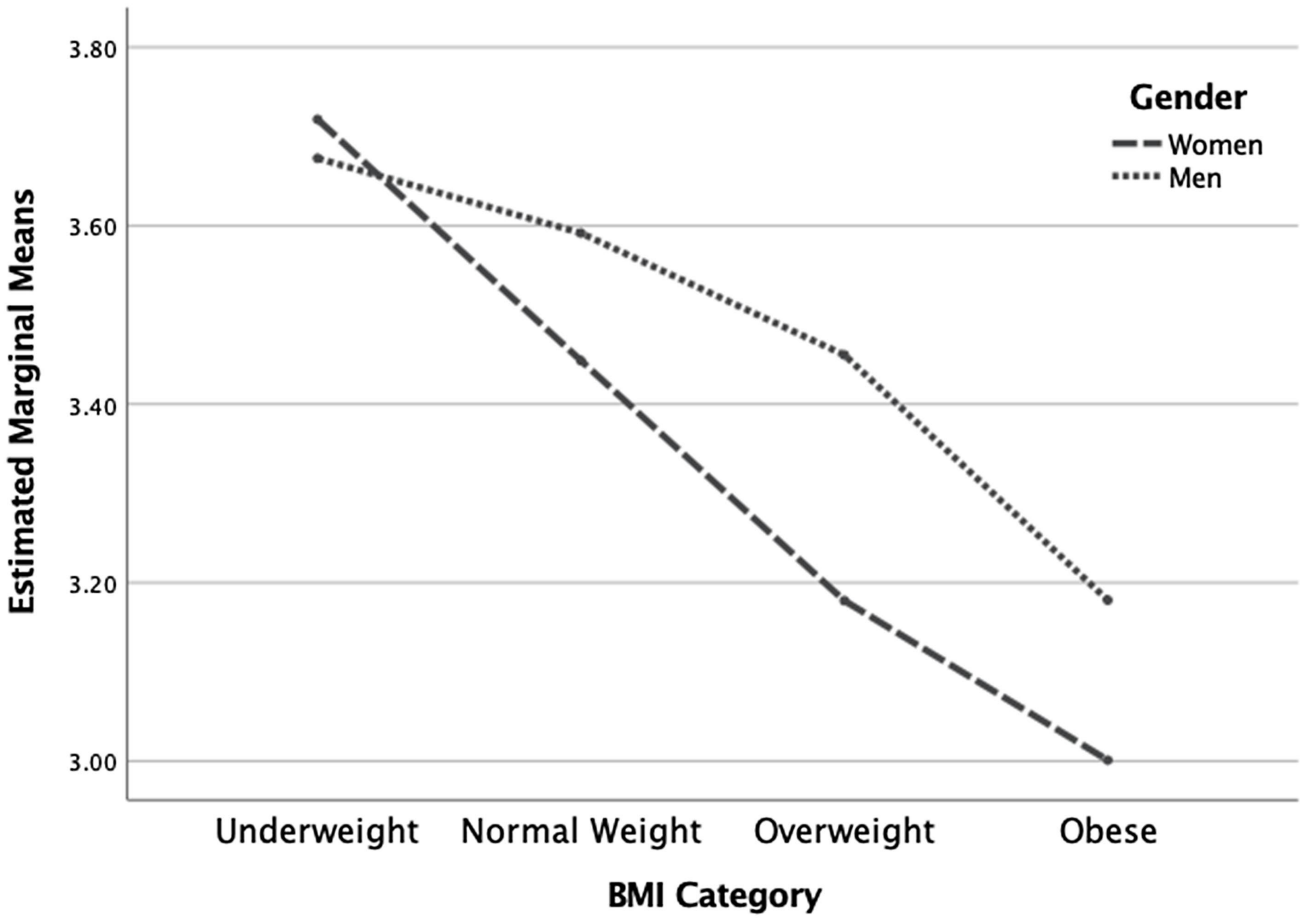
Interaction effect for intuitive eating total score. *N* = 1,312 (*n* = 657 for women, and *n* = 655 for men), *p* < 0.001.

**Figure 2:**
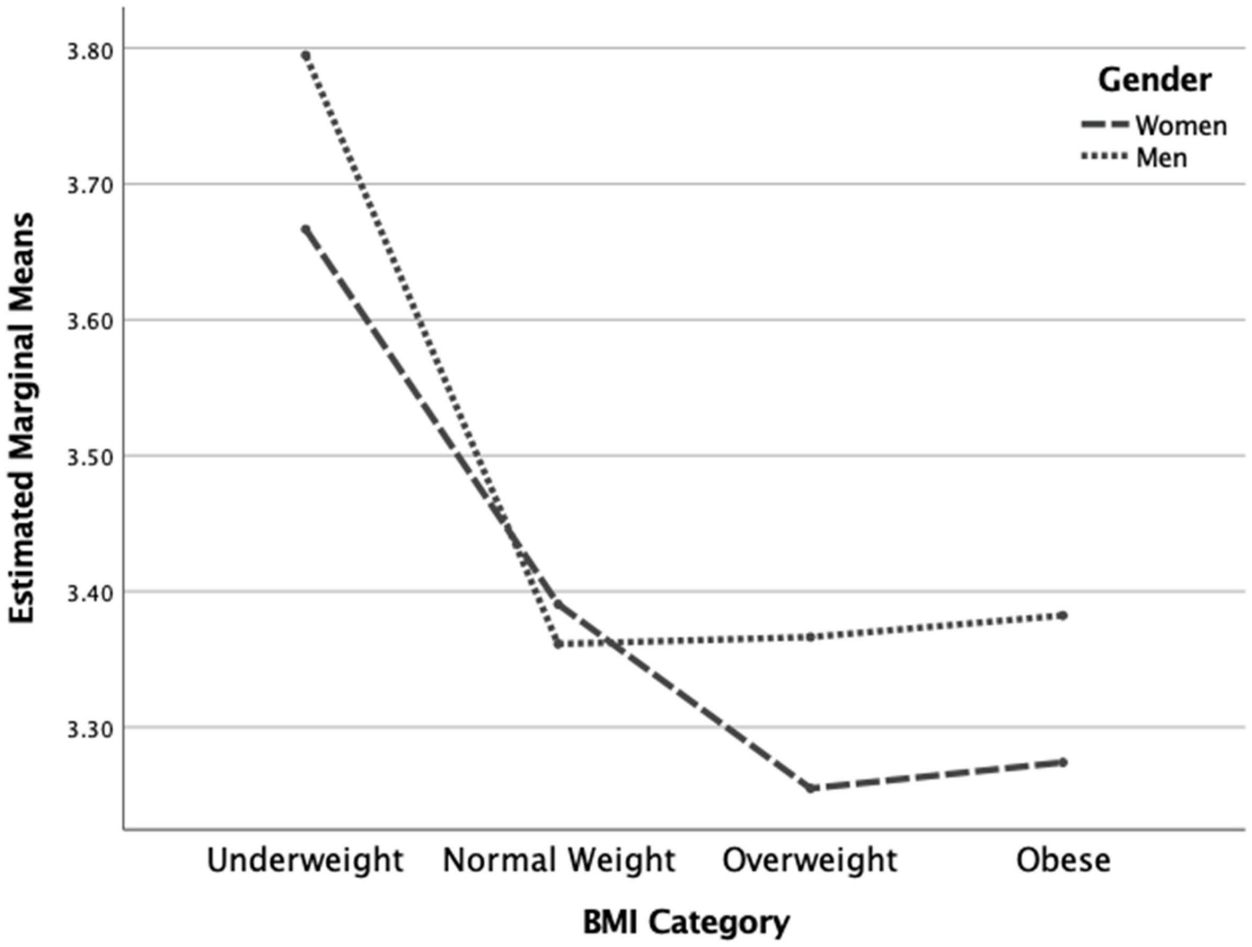
Interaction effect for unconditional permission to eat. *N* = 1,312 (*n* = 657 for women, and *n* = 655 for men), *p* = 0.01.

**Figure 3:**
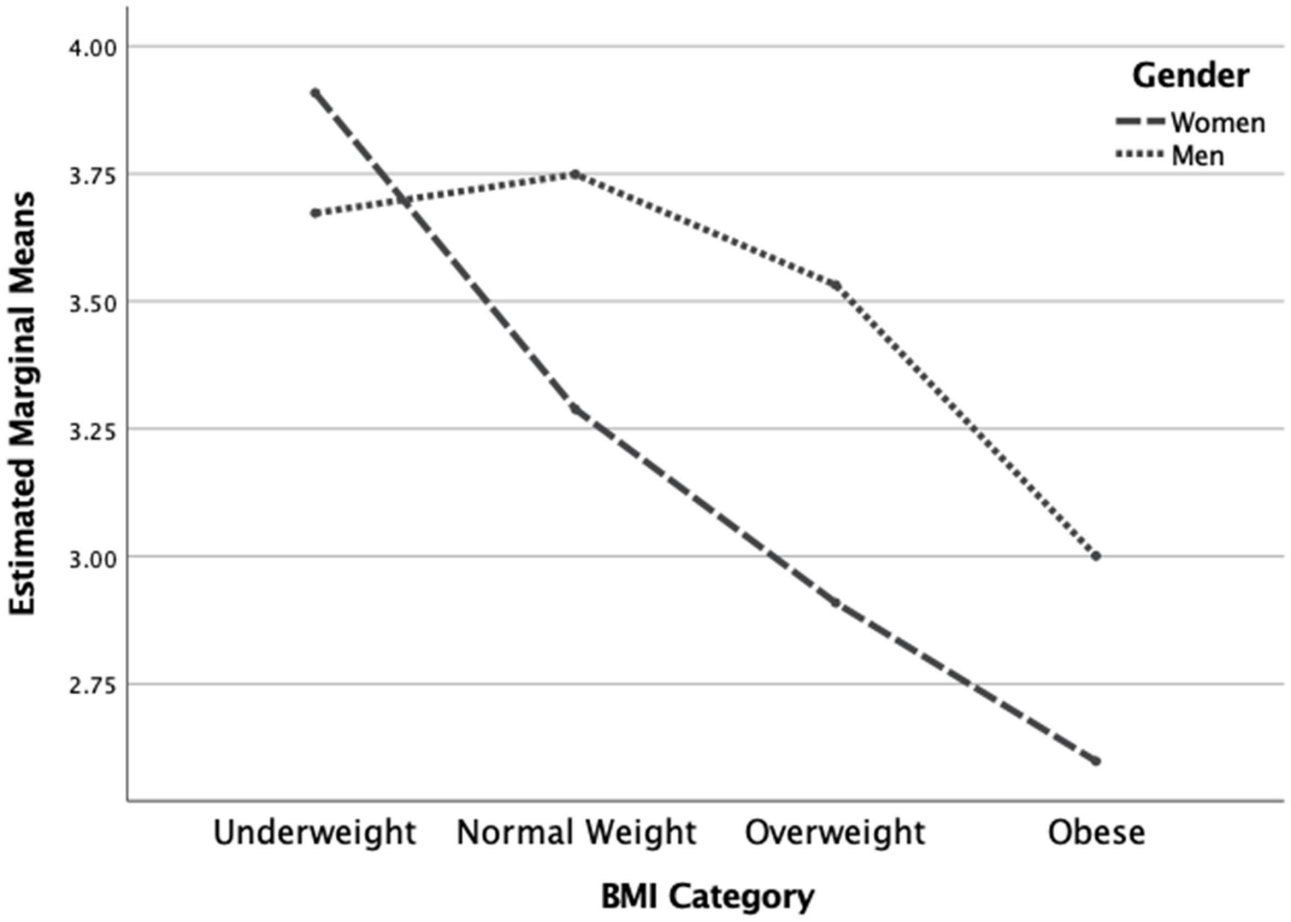
Interaction effect for eating for physical rather than emotional reasons. *N* = 1,312 (*n* = 657 for women, and *n* = 655 for men), *p* < 0.001.

**Figure 4:**
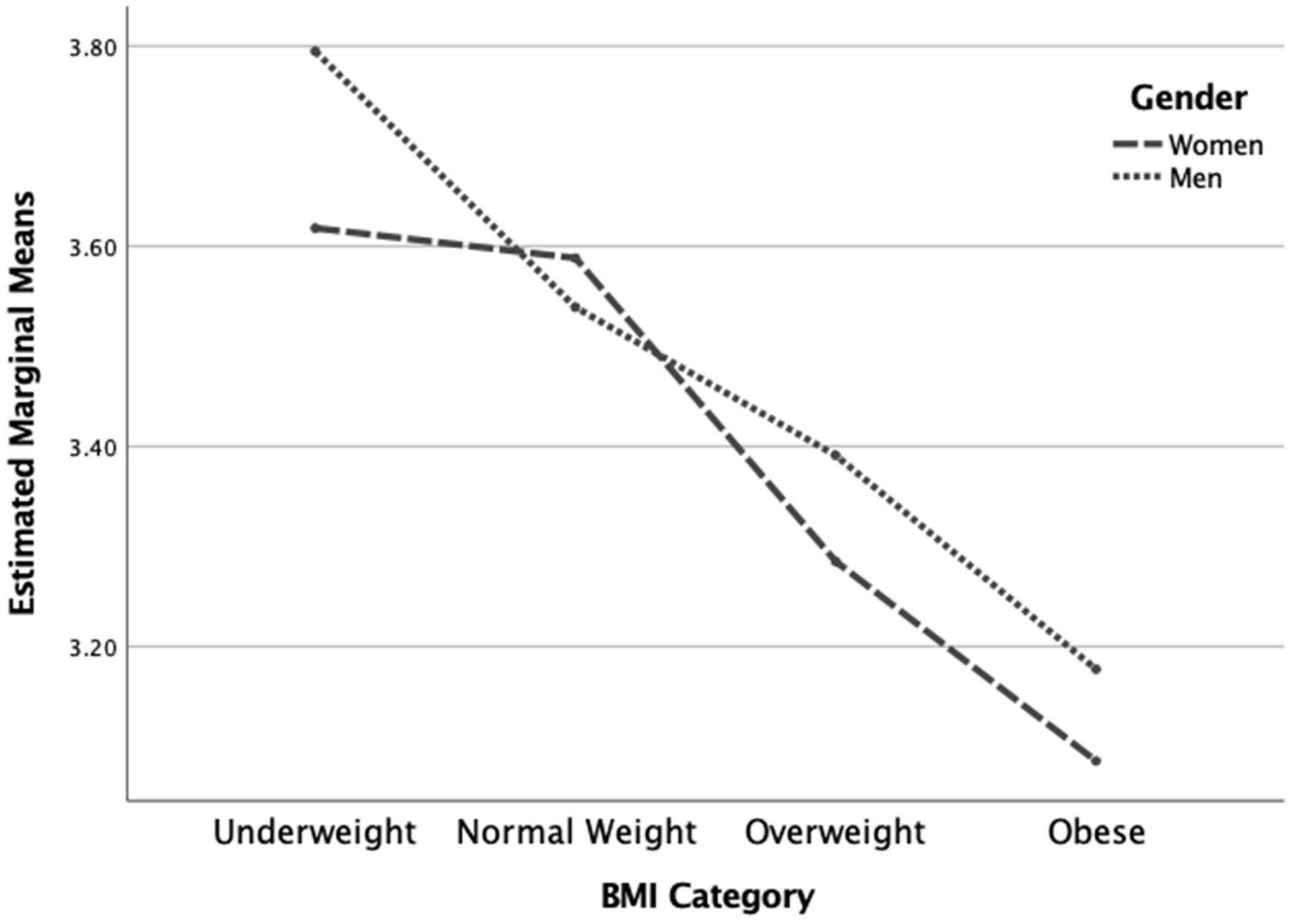
Interaction effect for reliance on hunger and satiety cues. *N* = 1,312 (*n* = 657 for women, and *n* = 655 for men), *p* < 0.001.

**Figure 5:**
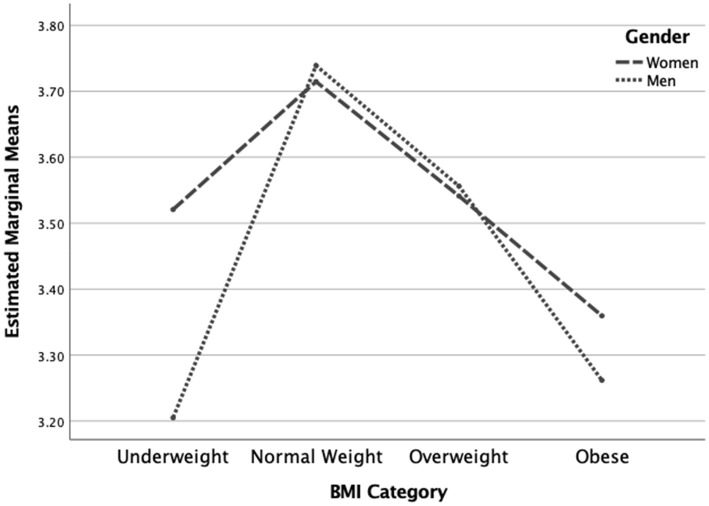
Interaction effect for body food choice congruence. *N* = 1,312 (*n* = 657 for women, and *n* = 655 for men), *p* < 0.001.

The two-way MANOVA revealed a statistically significant overall interaction effect for gender and BMI category on the dependent variables [*F*(7, 1,304) = 7.19, *p* < 0.001]. The interaction effect was also significant for each of the dependent variables (see Table 3). Concerning the IES total score, men had a trend of higher scores in all BMI categories except in the underweight category (see Figure 1). Concerning the Unconditional Permission to Eat scale, males had similar scores in all weight categories except in the underweight category where scores were higher. Additionally, females were decreasing in scores and plateaued at the higher weight categories, implying that males are less affected overall by this variable than females, regardless of weight category (See Figure 2). Concerning the Eating for Physical Rather than Emotional Reasons scale, both genders had the same pattern of decreasing levels as body weight increased. However, this is not the case in the underweight category where females have higher levels than males (see Figure 3). Concerning Hunger Satiety Cues, the same pattern was present with males have a steady drop in levels of hunger satiety cues as their weight increased whereas in females there are similar scores in the underweight and normal weight categories and then decrease in the higher weight categories (see Figure 4). Lastly, concerning the Body-Food Choice Congruence scale, both genders have the same overall pattern of lower levels in the underweight category, increasing in the normal weight category and dropping again in the overweight and obese categories. However, this pattern was evident in men who had lower levels than women in the underweight and obese categories (see Figure 5).

**Table 3:** Means and standard deviations for intuitive eating scores among BMI subgroups.

	Underweight		Normal weight		Overweight		Obese	
	Women (*n* = 48)	Men (*n* = 13)	Women (*n* = 416)	Men (*n* = 244)	Women (*n* = 117)	Men (*n* = 272)	Women (*n* = 76)	Men (*n* = 126)
IE	3.72 (0.50)	3.68 (0.37)	3.45 (0.56)	3.59 (0.47)	3.18 (0.57)	3.46 (0.48)	3.00 (0.57)	3.18 (0.43)
UPE	3.67 (0.85)	3.80 (0.89)	3.39 (0.71)	3.36 (0.74)	3.26 (0.64)	3.37 (0.64)	3.27 (0.65)	3.38 (0.65)
EPRTE	3.91 (0.75)	3.67 (0.55)	3.29 (0.90)	3.75 (0.79)	2.91 (0.96)	3.53 (0.85)	2.60 (1.00)	3.00 (0.84)
ROHSC	3.62 (0.75)	3.80 (0.83)	3.59 (0.74)	3.54 (0.78)	3.29 (0.78)	3.39 (0.67)	3.09 (0.83)	3.18 (0.67)
BFCC	3.52 (0.81)	3.21 (0.96)	3.72 (0.74)	3.74 (0.76)	3.54 (0.74)	3.56 (0.75)	3.36 (0.75)	3.26 (0.71)

*N* = 1,312. IE, intuitive eating; UPE, unconditional permission to eat; EPRTE, eating for physical rather than emotional reasons; ROHSC, reliance on hunger and satiety cues; BFCC, body-food choice congruence.

Concerning the main effects of gender, individual ANOVAs indicated a significant difference between males and females on the IES total Score [*F*(1,1,304) = 8.496, *p* = 0.004, *η^2^* = 0.006] and the Eating for Physical Rather than Emotional Reasons Subscale [*F*(1,1,304) = 15.008, *p* < 0.001, *η^2^* = 0.011]. In both cases, males scored significantly higher than females. No other gender differences were detected.

Concerning the main effects of BMI category, individual ANOVAs indicated a significant difference between the four BMI categories on all five scales (Table 4). Specifically, there was a significant main effect on the IES total Score [*F*(3,1,304) = 41.75, *p* < 0.001, *η^2^* = 0.088], the Unconditional Permission to Eat [*F*(3,1,304) = 4.67, *p* = 0.003, *η^2^* = 0.011], the Eating for Physical Rather than Emotional Reasons [*F*(3,1,304) = 38.68, *p* < 0.001, *η^2^* = 0.082], the Hunger Satiety Cues [*F*(3,1,304) = 20.65, *p* < 0.001, *η^2^* = 0.045] and the Body-Food Choice Congruence [*F*(3,1,304) = 17.16, *p* < 0.001, *η^2^* = 0.038]. In all four BMI categories, most scale scores significantly differed between them (*p*_’s_ of 0.03 to 0.000) with mean scores significantly decreasing as each BMI category increased, meaning that levels of total intuitive eating and its subscales were decreasing as body weight of participants increased. The only exceptions were found in: (a) the subscale of Unconditional Permission to Eat where individuals in the normal weight category did not significantly differ compared to the overweight and obese categories implying similar results between them (b) the Hunger Satiety Cues where individuals in the Underweight and Normal Weight categories had similar results and (c) the Body-Food Choice Congruence where individuals in the underweight category had similar results to the overweight and obese categories.

**Table 4:** Gender by BMI category interaction statistics on all variables of interest.

	*Mean square*	*F*	*P*
IE	5.75	21.92	<0.001
UPE	1.29	2.66	0.010
EPRTE	20.97	27.71	<0.001
ROHSC	5.19	9.54	<0.001
BFCC	4.60	8.19	<0.001

*N* = 1,312. IE, intuitive eating; UPE, unconditional permission to eat; EPRTE, eating for physical rather than emotional reasons; ROHSC, reliance on hunger and satiety cues; BFCC, body-food choice congruence.

## Discussion

The current study aimed to explore differences in intuitive eating scores based on gender and BMI in Greek-Cypriot adults. Overall, our findings suggest that both gender and body mass are important factors which influence intuitive eating levels in Greek-Cypriot adults. More specifically, we found that men generally tend to have higher levels of intuitive eating than women, and that body mass is inversely associated with intuitive eating for both genders. In addition, significant interaction effects between body mass and gender were observed.

The finding that men tend to eat more intuitively than women was expected, and is in line with previous studies with similar findings (Linardon et al., 2021). This finding may be explained in the context of the societal pressures that men and women face. The pressures placed on women to conform to beauty ideals and the degree to which their bodies are objectified and scrutinized tend to be greater than for men, with women experiencing greater body-surveillance, thin-ideal internalization, appearance-related media pressures, and family pressures (Grogan, 2016; Frederick et al., 2022b). In addition, recent work has shown women to be more likely to follow a weight loss diet or to attempt to control their appearance through crash diets/fasting (Frederick et al., 2022a). Consequently, women may be more likely to impose restrictions on their food consumption and be less likely to rely on their internal physiological hunger and satiety cues to guide their eating behavior.

It is worth noting that our results found intuitive eating to be inversely associated with BMI, which was also expected in light of previous work (Linardon et al., 2021). This finding might also be explained from a sociocultural perspective, and common attitudes held by many societies which equate having a lower BMI to enhanced health and wellbeing, and a higher BMI with unattractiveness and laziness, physically “unfit” or “inactive” (Puhl and Heuer, 2009). Such attitudes, which come to be internalized by individuals with a higher BMI, are related to body functionality and functionality appreciation (i.e., acknowledging and appreciating the functions the body performs; Alleva et al., 2017; Alleva and Tylka, 2021). Indeed, individuals with higher BMI have been found to have higher levels of functionality appreciation (Soulliard et al., 2019; Soulliard and Vander Wal, 2019; Todd et al., 2019). Therefore, it may be that individuals with a higher BMI tend to be less inclined to trust their body’s physiological hunger and satiety cues, thus prioritizing these in guiding their eating behavior over external emotional and situational cues.

We also found significant interaction effects between gender and body mass on intuitive eating. More specifically, the strongest interaction effects were observed for IES total scores and eating for emotional rather than physical reasons, with men in all BMI categories except in the underweight category scoring higher on both. Based on this finding, it seems that having a higher BMI is more likely to negatively affect intuitive eating in women than in men. Examining these interaction effects for the individual intuitive eating subscales provided further insights. First, the results showed that unconditional permission to eat in men is less likely to be affected by BMI than it is in women. Put differently, as body mass increases, women seem to be much more likely to pose restrictions on their diet than men. In addition, women who are in the underweight category seem to be significantly more likely to eat for physical reasons rather than emotional reasons, than men who are in the underweight category. When it comes to relying on hunger and satiety cues to guide eating behavior, women in the normal weight category tend to score higher than men, who have higher scores for all other categories (i.e., underweight, overweight, and obese). Finally, being underweight or being obese seems to be associated with lower levels of body food choice congruence in men and higher in women. Overall, these findings identify specific facets of intuitive eating which may be important to explore in targeted intervention plans aimed at promoting intuitive eating in Greek-Cypriot adults.

### Strengths and limitations

To the best of our knowledge, the current study was the first to compare levels of intuitive eating based on gender and body mass in the Greek Cypriot population. However, the findings should be considered in light of several limitations. Firstly, the correlational nature of the study design is somewhat limiting, as it does not allow for causal conclusions to be drawn. Secondly, the assessment of intuitive eating relies solely on self-report recall, which assumes participants’ accurate portrayal of their level of functioning and that their perception of their psychological functioning and eating behaviors are an accurate reflection of reality. For example, some studies found no relationship between self-report measures of dietary restraint behaviors and actual caloric intake over several weeks (Stice et al., 2004, 2010). This suggests that self-report dietary restraint scales often measure intentions or desires rather than actual behaviors. In addition, it cannot be ruled out that participants’ responses may have been affected by social and personal desirability, with participants giving responses according to how they want to feel or eat rather than how they actually do. Future studies would therefore do well to control for social desirability when examining intuitive eating using correlational designs.

The present work was also limited by the opportunistic sampling method and use of snowball sampling. For instance, the current study did not assess other variables which may influence eating styles (e.g., health-conditions, psychological distress, and type of diet). These factors may have had a confounding effect on the results, and future studies would do well to assess such additional variables.

## Data availability statement

The raw data supporting the conclusions of this article will be made available by the authors, without undue reservation.

## Author contributions

MA and EA: conceptualization, investigation, data curation, methodology, and writing, reviewing and editing. All authors contributed to the article and approved the submitted version.

## Conflict of interest

The authors declare that the research was conducted in the absence of any commercial or financial relationships that could be construed as a potential conflict of interest.

## Publisher’s note

All claims expressed in this article are solely those of the authors and do not necessarily represent those of their affiliated organizations, or those of the publisher, the editors and the reviewers. Any product that may be evaluated in this article, or claim that may be made by its manufacturer, is not guaranteed or endorsed by the publisher.

## Footnotes

^1^ www.qualtrics.com
